# Depth-dependent stabilization mechanisms of soil organic carbon and total nitrogen in different mixed modes of subtropical Moso bamboo forests

**DOI:** 10.3389/fmicb.2025.1671811

**Published:** 2025-11-12

**Authors:** Lingyuan Yan, Decai Gao, Huimin Wang, Shengwang Meng, Gang Lin, Jingying Fu

**Affiliations:** 1 Institute of Geographic Sciences and Natural Resources Research, Chinese Academy of Sciences, Beijing, China; 2 Qianyanzhou Ecological Research Station, Key Laboratory of Ecosystem Network Observation and Modeling, Institute of Geographic Sciences and Natural Resources Research, Chinese Academy of Sciences, Beijing, China; 3 Taihe-Qianyanzhou Ecological Research Center, Ji’an, Jiangxi, China; 4 College of Resources and Environment, University of Chinese Academy of Sciences, Beijing, China

**Keywords:** Moso bamboo, soil microorganisms, enzyme activity, mixed-species forests, soil organic carbon

## Abstract

Forest soils play a pivotal role in terrestrial carbon (C) sequestration and nitrogen (N) cycling, particularly in subtropical Moso bamboo (*Phyllostachys edulis*) forests ecosystems. While prior studies have explored soil organic carbon (SOC) and total nitrogen (TN) dynamics in bamboo systems, the depth-dependent stabilization mechanisms governing these stocks under contrasting mixed-species regimes remain unresolved, limiting predictions of long-term C/N storage. Here, we investigated SOC and TN in stratified soil samples (0–100 cm) across three forest types in southeastern China: pure Moso bamboo (Mb), mixed Moso bamboo-evergreen broadleaved (MbB), and mixed Moso bamboo-Chinese fir (*Cunninghamia lanceolata*) (MbF) forests. Results showed that SOC and TN stocks showed no significant differences between MbB and Mb across all soil layers (0–20, 20–40, 40–60, 60–80, and 80–100 cm) or within the entire 0–100 cm soil profile. While soils (0–100 cm) in MbB exhibited enhanced enzyme activity (*β*-glucosidase: +52%; N-acetyl-glucosaminidase: +89%) and ammonium availability (+47%) compared to Mb, equivalent SOC and TN stocks across 0–100 cm profiles revealed microbial priming effects and stoichiometric constraints offsetting litter-derived C gains. In contrast, MbF displayed substantial TN depletion (−32% vs. Mb) across the entire 0–100 cm soil profile with parallel SOC/TN reductions in subsurface layers (20–40 cm: −42% SOC, −48% TN), driven by coniferous lignin inputs and microbial N mining, no significant differences were detected in the 0–20, 40–60, 60–80 or 80–100 cm layers. Vertical stratification analysis demonstrated shifting regulatory controls: microbial biomass dominated surface SOC/TN stabilization, while inorganic N dynamics and enzymatic activities controlled deeper horizons. These findings establish that SOC stability emerges from depth-specific enzyme-microbe-mineral interactions, while TN stocks reflect microbial stoichiometric adaptation to litter chemistry - critical insights for optimizing mixed-species strategies in bamboo forest management.

## 1 Introduction

Moso bamboo (*Phyllostachys edulis*) forests, covering over 5 million hectares in subtropical China, exhibit exceptional carbon (C) sequestration capacity (6.8–7.3 t C ha^−1^ yr.^−1^) through vegetation and soil mechanisms, surpassing other regional forest types (Chen et al., 2009; Yuen et al., 2017; Song et al., 2020; IPCC, 2022; Lv et al., 2024). Soil nitrogen (N) pools—critical regulators of C cycling and ecosystem productivity—interact closely with bamboo’s growth and C sequestration processes: their extensive rhizome networks and rapid root turnover drive substantial soil organic carbon (SOC) and total nitrogen (TN) accumulations through exudates and necromass inputs (Song et al., 2016; Xu et al., 2023). Critically, soil N availability serves as a key regulator of ecosystem productivity and C cycling processes, constraining both plant growth and microbial decomposition (Janssens et al., 2010; Xu et al., 2020). However, monoculture practices often lead to soil acidification and reduced microbial diversity, which not only impair soil N transformation (e.g., mineralization, nitrification) but also threaten long-term C storage—exacerbating the interdependence between soil N pool functionality and ecosystem C sequestration (Yang et al., 2019; Qiao et al., 2020). Mixed-species systems (e.g., bamboo with Chinese fir or broadleaved trees) may enhance SOC stability through diversified litter inputs and improved soil enzyme activity (Chen et al., 2018; Zhang M. et al., 2024, Zhang X. et al., 2024), and such diversification could further optimize soil N pool dynamics (e.g., balancing N supply and demand, promoting N retention) to sustain high ecosystem productivity; yet the mechanisms underlying these benefits remain unclear. Understanding these interactions is critical for sustainable management of these high-productivity ecosystems.

Studies focusing on pure Moso bamboo forests have consistently reported high SOC and TN accumulation in surface soils, largely driven by rapid root turnover and rhizodeposition (Yen, 2015; Xu et al., 2023). However, under monoculture, these systems often experience declining pH and reduced fungal diversity, which can limit the long-term stability of C pools (Yang et al., 2019; Luo et al., 2024). In comparison, mixed Moso bamboo forests show divergent outcomes, with studies reporting positive, neutral, or even negative effects (Conti and Diaz, 2013; Dawud et al., 2017; Chen et al., 2018; Chen et al., 2023). Emerging evidence suggests that mixed-species plantations may enhance SOC accumulation through: (1) diversified litter inputs stimulating enzyme activity, and (2) increased microbial necromass formation (Lange et al., 2015). For Moso bamboo mixed forests, reports on their influence on SOC and TN stocks vary widely, ranging from significant enrichment to negligible or even negative impacts (Jin et al., 2023; Sardar et al., 2023; Liu et al., 2025). The mechanisms governing SOC and N dynamics remain unresolved. Given bamboo’s unique rhizosphere properties—high root turnover but low lignin input—interactions with broadleaf or conifer species may differentially influence microbial processing and C stabilization. Resolving these interactions is critical for predicting the C sequestration potential of managed bamboo-tree systems.

Beyond horizontal comparisons, the vertical distribution of SOC and TN throughout the soil profile plays a critical role in the long-term sequestration potential of bamboo ecosystems (Qiao et al., 2020; Huang et al., 2023). As a significant C sink in subtropical China, Moso bamboo forest ecosystems have been the focus of growing research into mixed-species regimes. However, findings regarding their effects on SOC and TN stocks remain inconsistent, with studies reporting outcomes ranging from significant enrichment to negligible or even negative impacts (Jin et al., 2023; Sardar et al., 2023; Liu et al., 2025). Much of the existing work has concentrated on shallow soil layers (0–60 cm), consistently documenting vertical declines in SOC and TN concentrations with depth (Yang et al., 2019; Zhang M. et al., 2024, Zhang X. et al., 2024). This emphasis on surface soils has limited our understanding of the contributions of subsoil layers (> 60 cm) to total ecosystem C and N stocks—especially in mixed bamboo-tree systems, where management practices may distinctly alter deeper soil processes. The vertical distribution of SOC and TN is strongly mediated by enzyme activity, with distinct stratification patterns observed for microbial biomass and extracellular enzymes (Bardgett and Van Der Putten, 2014). Diverse tree communities promote mineral-associated organic C formation via fungal-driven decomposition and reduced respiratory losses (Clemmensen et al., 2013; Chen et al., 2022). Despite representing a small fraction of total SOC (< 5%), microbial biomass serves as both a rapid-cycling C pool and key mediator of nutrient transformations (Weverka et al., 2023). However, these mechanisms remain poorly characterized in bamboo-dominated systems, particularly across the full soil profile. Understanding these depth-dependent microbial processes is essential for predicting long-term C sequestration potential in managed bamboo forests.

Here, through a field investigation, we systematically examined how tree species mixing and vertical soil stratification influence the storage and distribution patterns of SOC and TN pools. Our objectives were: (1) to identify the mechanisms through which tree diversity modulates SOC and TN accumulation, and (2) to determine how soil depth mediates the vertical stratification of SOC/TN stocks and their regulatory drivers. We hypothesized that (1) mixed forests (particularly bamboo–evergreen broadleaved systems) would accumulate greater SOC and TN stocks than monocultures due to complementary litter chemistry and root exudation patterns (Lyu et al., 2023; Li et al., 2024); (2) forest-type influences would dominate surface layers (0–20 cm) through biotic controls (litter inputs, rhizosphere activity), while geochemical factors (mineral surface area, iron oxides) would increasingly govern SOC/TN stabilization below 20 cm (Goebes et al., 2019).

## 2 Materials and methods

### 2.1 Site description

The study was conducted in Le’an County, Jiangxi Province, southern China (26°50′-27°45′N, 115°35′-116°10′E), a region characterized by a subtropical humid monsoon climate with pronounced seasonal variability in temperature and precipitation. The mean annual temperature is 18.2 °C (ranging from −8 °C to 40.7 °C), and the annual precipitation is 1757.7 mm, with ~60% concentrated between April and June. The area receives 1557.7 h of annual sunshine and has a forest coverage of 70.23%, of which bamboo forests constitute > 14% of the total land area. The native vegetation consists of evergreen broadleaved forests, while plantation forests are dominated by Masson pine (*Pinus massoniana*), slash pine (*Pinus elliottii*), and Chinese fir (*Cunninghamia lanceolata*).

### 2.2 Experimental design

In June 2024, we established 12 permanent sampling plots (each 30 m × 30 m), representing four forest types with three independent replicates each in a subtropical region of China. The forest types were: (1) pure Moso bamboo forests (*Phyllostachys edulis*; Mb), (2) mixed Moso bamboo–evergreen broadleaved forests (MbB), the dominant evergreen broadleaved species included *Castanopsis sclerophylla*, *Quercus glauca* and (3) mixed Moso bamboo-Chinese fir forests (MbF). The use of three replicated plots per forest type follows common practices in forest ecology and provides a robust basis for statistical analysis. To control for environmental variability, all study sites were selected with similar slope gradients, aspect, and soil parent material. Within each plot, we established five equidistant sampling points along an “S”-shaped transect to ensure spatial representativeness. At each sampling point, five soil cores were extracted using a stainless-steel auger (5 cm diameter) and composited into one representative sample per point to minimize small-scale heterogeneity. Soil collection followed a stratified depth approach across five vertical layers: 0–20 cm, 20–40 cm, 40–60 cm, 60–80 cm, and 80–100 cm. The cores from the same depth interval at each point were combined to form a composite sample for that depth. Fresh soil samples were immediately placed in sterile polypropylene containers and stored on dry ice during field transportation. Within 6 h of collection, samples were homogenized by sieving through a 2-mm stainless steel mesh to remove root fragments and gravels. Processed samples were stored at 4 °C in dark conditions prior to laboratory analyses, with all biochemical assays completed within 72 h of sampling. For SOC and TN determination, a separate subsample was air-dried at ambient temperature (≈ 25 °C) for 14 d in a clean, ventilated room. After reaching constant mass, the air-dried soil was gently crushed and passed through a 2-mm sieve; a further aliquot was ground to < 0.15 mm (100 mesh) to ensure homogeneity.

### 2.3 Soil analysis

#### 2.3.1 Soil organic carbon and total nitrogen

Soil organic carbon content was tested by the H_2_SO_4_–K_2_Cr_2_O_7_ method; soil total nitrogen was determined by the Kjeldahl determination method (Bao, 2000).

The SOC and TN stocks (kg m^−2^) were calculated as follows by the previous methods (Chen et al., 2023):

$${\mathrm{SOC}}_{\text{iStock}}=\mathrm{SO}C_{i}\times BD_{i}\times D_{i}\times 100$$

$${\mathrm{TN}}_{\text{iStock}}=TN_{i}\times BD_{i}\times D_{i}\times 100$$

Where *SOC_i_* is the soil organic carbon content of the i-th soil layer (g kg^−1^), *TN_i_* is the total nitrogen concent of the i-th soil layer (g kg^−1^), *BD_i_* is the bulk density of the i-th soil layer (g cm^−3^), and *D*_i_ is the depth of the i-th soil layer (cm). *BD* for each depth layer was determined using the standard core method. Briefly, stainless steel cutting rings (5 cm in height and 5 cm in diameter) were used to collect undisturbed soil cores from the wall of each soil profile. The samples were then oven-dried at 105 °C for 48 h to obtain the dry mass. Bulk density was calculated as the mass of dry soil per unit volume (g cm^−3^). These measured *BD* values were subsequently used to calculate the SOC and TN stocks for each soil layer.

#### 2.3.2 Soil ammonium and nitrate nitrogen

Soil ammonium nitrogen (NH_4_^+^-N) and nitrate nitrogen (NO_3_^−^-N) concentrations were determined using the indophenol blue method (Kempers and Zweers, 1986) and the phenolate disulphonic acid method (Cataldo et al., 1975), respectively. Specially, fresh soil samples (5.0 ± 0.1 g) were extracted with 25 mL 2 M KCl (1:5 w/v ratio) by shaking (180 rpm, 1 h, 25 °C). After centrifugation (4,000 × g, 10 min), supernatants were filtered (0.45 μm cellulose acetate) and analyzed colorimetrically. Finally, the concentration was measured using a visible light photometer.

#### 2.3.3 Soil extracellular enzymatic activities

We measured *β*-glucosidase (BG, EC 3.2.1.21) and N-acetyl-glucosaminidase (NAG, EC 3.2.1.30) activities using fluorogenic 4-methylumbelliferyl substrates following Marx et al. (2001) with modifications by German et al. (2011). In brief, soil suspensions were prepared by mixing 2.00 g of fresh soil (sieved at 2 mm) with 100 mL of sodium acetate buffer (pH = 5.0) and stirring for 1 min using a magnetic stir plate. Assay 96-well microplates were prepared by adding 200 μL of soil suspensions and 50 μL of substrates. For the blank 96-well microplates, 200 μL of soil suspensions and 50 μL of sodium acetate buffer (pH = 5.0) were added. Quench 96-well microplates were set up by combining 200 μL of soil suspensions with 50 μL of 4-methylumbelliferyl. Lastly, standard 96-well microplates were prepared by mixing 200 μL of sodium acetate buffer (pH = 5.0) with 50 μL of 4-methylumbelliferyl. Subsequently, the 96-well microplates were incubated in the dark at 25 °C for a duration of 4 h. Upon completion of the incubation, 10 μL of 0.5 mol L^−1^ NaOH was swiftly introduced into all 96-well microplates to cease the reaction process. Finally, the fluorescence products were promptly assessed using a fluorimetric microplate reader (Synergy H1, BioTek, Winooski, VT), with excitation and emission wavelengths set at 365 nm and 450 nm, respectively. The calculation of soil EEAs was performed following the methodology outlined by German et al. (2011).

#### 2.3.4 Soil microbial biomass

Soil microbial biomass carbon (MBC) and nitrogen (MBN) were measured using the chloroform fumigation extraction method, as described by Brookes et al. (1985) and Vance et al. (1987). Paired samples (fumigated for 24 h with CHCl₃ vs. unfumigated controls) were extracted with 0.5 M K₂SO₄ (1:4 w/v, 1 h shaking at 200 rpm). Extracts were analyzed for dissolved organic carbon (DOC) and total dissolved nitrogen (TDN) using a TOC/TN analyzer (Multi N/C 3100, Analytik Jena). MBC and MBN were calculated as:

MBC=EC/0.45

MBN=EN/0.54

Where EC represents the difference in DOC content between fumigated and unfumigated soil; EN represents the difference in TDN content between fumigated and unfumigated soil; the correction factors for the incomplete extraction of MBC and MBN are 0.45 and 0.54, respectively (Brookes et al., 1985; Vance et al., 1987).

### 2.4 Statistical analyses

The impacts of forest types (pure Moso bamboo forests, mixed Moso bamboo–evergreen broadleaved forests and mixed Moso bamboo-Chinese fir forests) on inorganic N availability (NH₄^+^-N and NO₃^−^-N concentrations), extracellular enzymatic activities (BG, NAG), and microbial biomass (MBC, MBN) and SOC and TN stocks were analyzed using one-way ANOVA (Least significant difference’s test at (*p* < 0.05). The statistical analyses were carried out in SPSS 26.0 (SPSS Inc., IL, USA). Pearson correlations analysis method was used to deal with the relationships among soil physicochemical properties, element and their stoichiometry. To analyze drivers of SOC and TN stocks across forest types and soil depths (0–100 cm), structural equation modeling was applied. Standardized path coefficients (*β*) quantified direct and indirect effects of predictors. The model fit was considered good using the Goodness-of-Fit Index (GFI ≥ 0.9), the Standardized Root Mean Square Residual (SRMR < 0.08), and the *χ*^2^ test (*p* > 0.05) (Liu M. et al., 2019, Liu X. et al., 2019; Chen et al., 2023). The SEM analyses were conducted using AMOS 28.0 (IBM SPSS Inc., Chicago, IL, USA).

## 3 Results

### 3.1 Soil organic carbon and total nitrogen stocks

**Figure 1:**
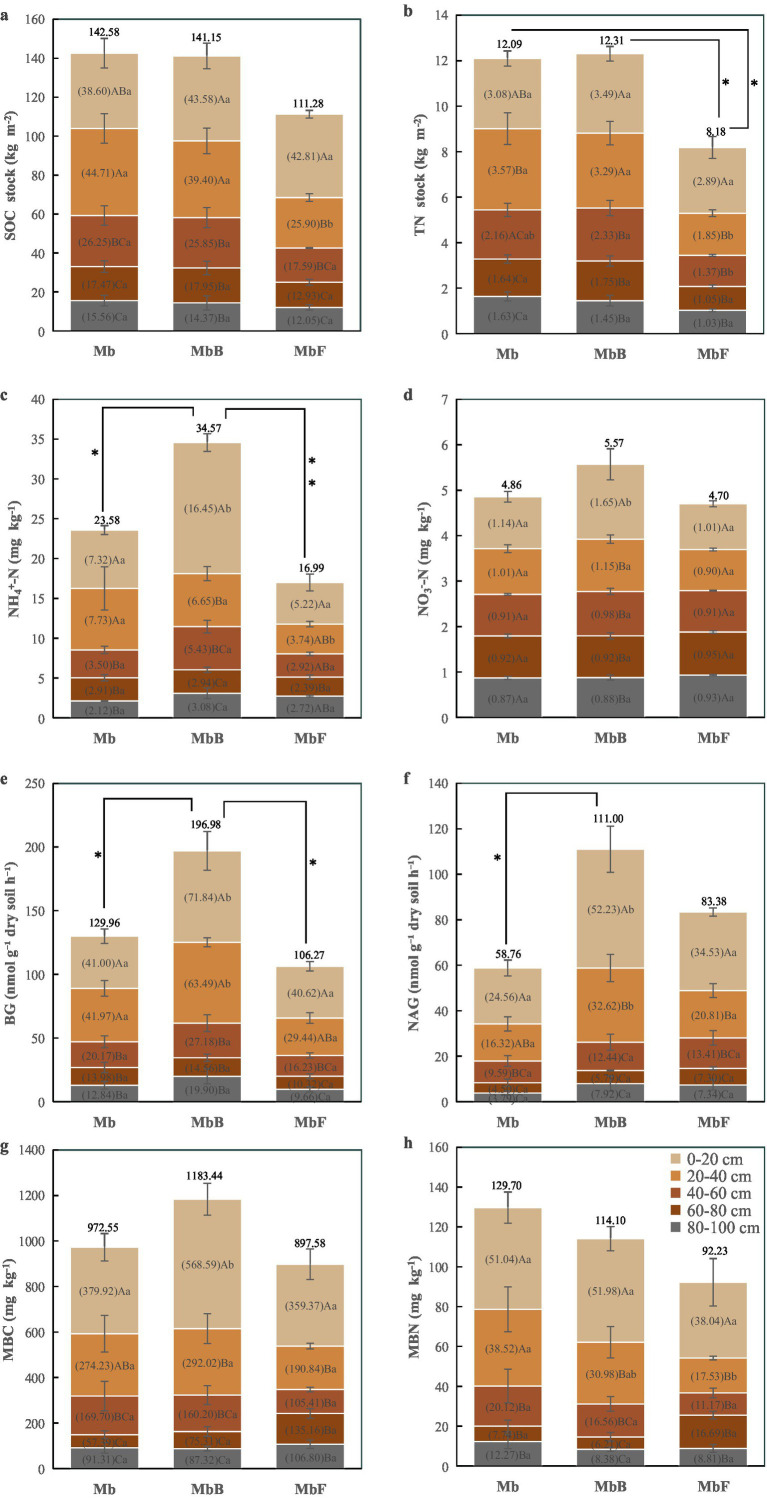
Vertical distribution of multiple soil indicators in three forest types. Soil organic carbon (SOC) **(a)** and total nitrogen (TN) **(b)** stocks (kg m^−2^), soil ammonium nitrogen (NH_4_^+^-N) **(c)** and nitrate nitrogen (NO_3_^−^-N) **(d)** concentrations (mg kg^−1^), soil *β*-glucosidase (BG) **(e)** and N-acetyl-glucosaminidase (NAG) **(f)** activities (nmol g^−1^ dry soil h^−1^), soil microbial biomass carbon (MBC) **(g)** and soil microbial biomass nitrogen (MBN) **(h)** concentrations (mg kg^−1^) across the 0–100 cm soil profile in three forest types: the pure Moso bamboo forest (Mb), the mixed Moso bamboo–evergreen broadleaved forest (MbB), and the mixed Moso bamboo-Chinese fir forest (MbF). Values shown within each panel represent the mean content for the corresponding soil layer (0–20, 20–40, 40–60, 60–80, and 80–100 cm). The values presented above each panel indicate the mean content for the entire 0–100 cm soil profile. Within each forest type, different uppercase letters **(a–c)** indicate significant differences (*p* < 0.05) among depth intervals. Lowercase letters **(a,b)** denote significant differences (*p* < 0.05) within the same layers. Error bars represent standard errors of the mean (*n* = 4). *indicates signifcant (*p* < 0.05), **indicates highly signifcant (*p* < 0.01).

Integrated analysis of the 0–100 cm soil profile showed no significant differences in SOC stocks among forest types (*p* > 0.05; Figure 1a). In contrast, TN stocks in MbF were significantly lower than those in Mb and MbB by 32 and 34%, respectively (*p* < 0.05; Figure 1b). This depletion was most pronounced in the 20–40 cm layer, where MbF exhibited 42 and 48% reductions in SOC and TN stocks relative to Mb (*p* < 0.05). No significant differences in SOC or TN stocks were observed between MbB and Mb.

Across all forest types, SOC and TN stocks generally declined with soil depth. However, Mb displayed a distinct vertical distribution pattern, with significantly higher SOC and TN stocks in the 20–40 cm layer compared to both overlying (0–20 cm) and underlying horizons (40–60 cm, 60–80 cm, and 80–100 cm; *p* < 0.05). This contrasted with the continuous decreases observed in MbB and MbF. In MbB, surface layers (0–20 cm and 20–40 cm) retained significantly higher SOC and TN stocks than deeper strata (40–100 cm; *p* < 0.05). In contrast, MbF exhibited peak C and N accumulation in the 0–20 cm layer, with significantly lower stocks in all subsequent depths (20–100 cm; *p* < 0.05).

### 3.2 Soil ammonium nitrogen and nitrate nitrogen

Significant variations in soil NH₄^+^-N concentrations were observed among forest types (Figure 1c). MbB showed 47% higher NH₄^+^-N concentrations than Mb throughout the 0–100 cm profile (*p* < 0.05), with a particularly marked increase of 125% in the surface layer (0–20 cm; *p* < 0.05). While MbF exhibited no overall differences in NH₄^+^-N concentrations across the full profile, it demonstrated a 52% reduction in the 20–40 cm layer compared to Mb (*p* < 0.05). In contrast, NO₃^−^-N concentrations showed minimal variation among forest types (Figure 1d). No significant differences were detected across the entire profile or within most depth increments, except for a 44% higher NO₃^−^-N concentration in MbB’s 0–20 cm layer relative to Mb (*p* < 0.05).

NH₄^+^-N concentrations generally declined with depth, with notable exceptions in Mb’s 20–40 cm layer and the deepest layers (80–100 cm) of MbB and MbF. MbB’s surface layer (0–20 cm) contained significantly higher concentrations of both N forms than deeper layers (*p* < 0.05). In Mb, NH₄^+^-N concentrations showed no significant differences between 0–20 cm and 20–40 cm layers, while MbF exhibited similar concentrations across its upper three layers (0–60 cm). NO₃^−^-N concentrations remained consistent across all layers in both Mb and MbF.

### 3.3 Microbial activity

The MbB demonstrated significantly higher extracellular enzyme activities compared to Mb (Figures 1e,f). Across the 0–100 cm profile, MbB showed 52% greater BG activity and 89% higher NAG activity than Mb (*p* < 0.05). These enhancements were most pronounced in surface layers, with 75% (BG) and 113% (NAG) increases in the 0–20 cm layer, and 51% (BG) and 100% (NAG) increases in the 20–40 cm layer (*p* < 0.05). No significant differences were observed between Mb and MbF.

Both BG and NAG activities exhibited a general decreasing trend with soil depth, with three exceptions: (1) elevated BG activity in Mb’s 20–40 cm layer, (2) increased BG and NAG activities in MbB’s 80–100 cm layer, and (3) higher NAG activity in Mb’s 80–100 cm layer. Below 40 cm depth, enzymatic activities remained statistically similar across all forest types, with no significant differences observed among the 40–60 cm, 60–80 cm, and 80–100 cm layers.

No significant differences in MBC or MBN were observed across the full 0–100 cm profile among forest types (FIgures 1g,h). However, vertical stratification revealed distinct patterns in specific layers. In surface layer (0–20 cm), MbB exhibited 50% higher MBC than Mb (*p* < 0.05), while no significant difference was detected between Mb and MbF. In subsurface layer (20–40 cm), MbF showed 54% lower MBN compared to Mb (*p* < 0.05), whereas MbB did not differ significantly from Mb.

Both MBC and MBN generally declined with depth across all forest types, with two exceptions: an increase in MBC and MBN in the 80–100 cm layer of Mb and MbB; elevated MBN in the 60–80 cm layer of MbF. Within the 0–20 cm layer, MbB and MbF maintained significantly higher MBC and MBN than deeper strata (*p* < 0.05), whereas Mb showed no difference between 0–20 cm and 20–40 cm. Below 40 cm, MBC and MBN were statistically indistinguishable among forest types (40–60 cm, 60–80 cm, and 80–100 cm).

### 3.4 Relationships between soil organic carbon, total nitrogen and microbial parameters

According to the Pearson’s correlation analysis (Supplementary Table S2), it showed that SOC was highly positively correlated with TN (r = 0.964, *p* < 0.01), MBC (r = 0.839, *p* < 0.01), MBN (r = 0.838, *p* < 0.01) and BG (r = 0.766, *p* < 0.01), and negatively correlated with soil depth (r = −0.761, *p* < 0.01). TN was highly positively correlated with MBN (r = 0.834, *p* < 0.01), MBN (r = 0.815, *p* < 0.01), and BG (r = 0.768, p < 0.01), and negatively correlated with soil depth (r = −0.690, *p* < 0.01, Supplementary Table S2). MBC correlated most closely with MBN (r = 0.945, *p* < 0.01).

### 3.5 Drivers of soil organic carbon and total nitrogen stocks in different forest types

Structural equation models (SEM) revealed key drivers of SOC and TN stocks across forest types, with all models showing good fit (GFI > 0.97, SRMR ≤ 0.032, *p* > 0.05).

**Figure 2:**
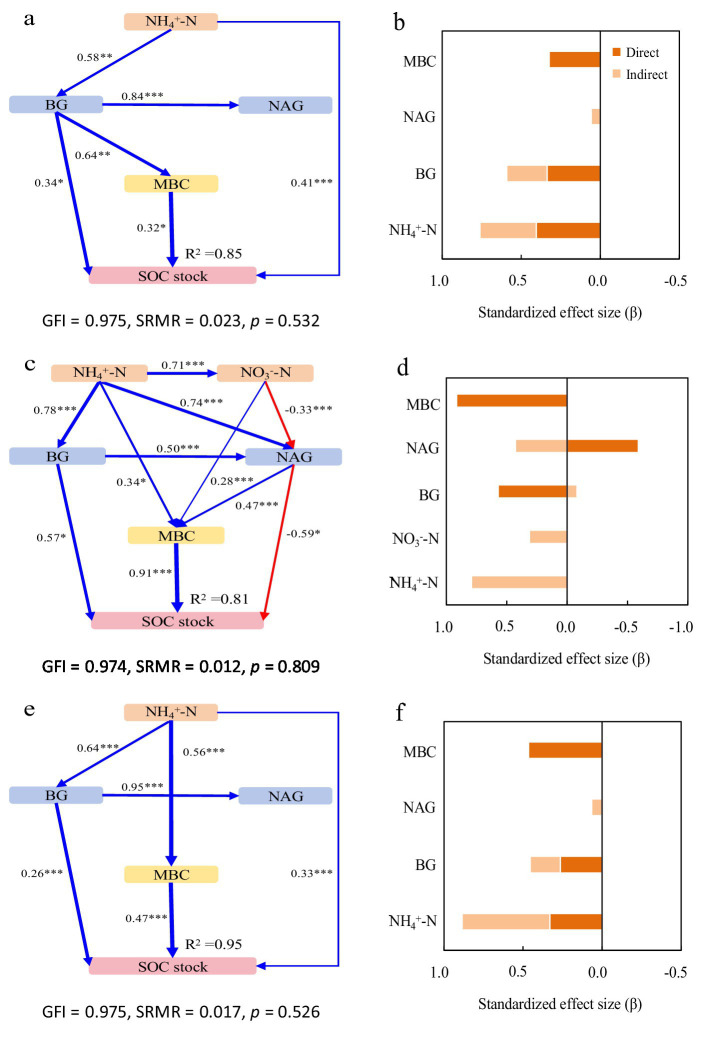
Structural equation models depicting the drivers of soil organic carbon (SOC) stocks in the pure Moso bamboo forest **(a,b)**, the mixed Moso bamboo–evergreen broadleaved forest **(c,d)**, and the mixed Moso bamboo-Chinese fir forest **(e,f)**. Explanatory variables include ammonium nitrogen (NH_4_^+^-N), nitrate nitrogen (NO_3_^−^-N), β-glucosidase (BG) and N-acetyl-glucosaminidase (NAG), microbial biomass carbon (MBC) and microbial biomass nitrogen (MBN). **(a,c,e)** Pathways analysis of SOC stocks. The figures adjacent to the arrows represent path coefficients and their corresponding *p* values. The symbols *, **, and *** denote significance levels of *p* < 0.05, *p* < 0.01, and *p* < 0.001, respectively; gray dashed arrows denote non-significant correlations (*p* > 0.05). **(b,d,f)** Summed the direct and indirect effects (Bars are not stacked, adjacent bars represent distinct effect types).

**Figure 3:**
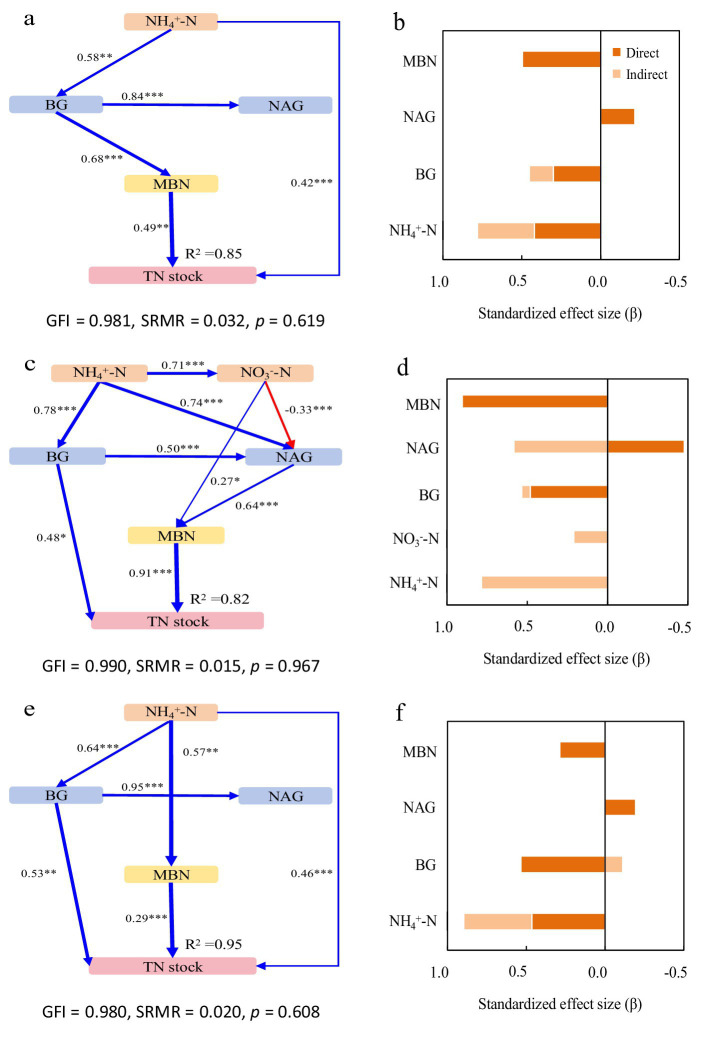
Structural equation models depicting the drivers of total nitrogen (TN) stocks in the pure Moso bamboo forest **(a,b)**, the mixed Moso bamboo–evergreen broadleaved forest **(c,d)**, and the mixed Moso bamboo-Chinese fir forest **(e,f)**. Explanatory variables include ammonium nitrogen (NH_4_^+^-N), nitrate nitrogen (NO_3_^−^-N), β-glucosidase (BG) and N-acetyl-glucosaminidase (NAG), microbial biomass carbon (MBC) and microbial biomass nitrogen (MBN). **(a,c,e)** Pathways analysis of TN stocks. The figures adjacent to the arrows represent path coefficients and their corresponding *p* values. The symbols *, **, and *** denote significance levels of *p* < 0.05, *p* < 0.01, and *p* < 0.001, respectively. **(b,d,f)** Summed the direct and indirect effects (Bars are not stacked, adjacent bars represent distinct effect types).

In Mb, the model explained 85% of the variance in both SOC and TN stocks (Figures 2a, 3a). NH₄^+^-N was the primary driver, exhibiting significant direct positive effects, with additional mediation through BG activity and microbial biomass. BG activity showed both direct and indirect positive effects, while NAG activity had contrasting impacts: indirect positive effects on SOC through MBC but direct negative effects on TN (Figures 2b, 3b).

In MbB, the model explained 81 and 82% of the variance in SOC and TN stocks, respectively (Figures 2c, 3c). Soil inorganic N (NH₄^+^-N and NO₃^−^-N) influenced stocks solely through enzymatic and microbial pathways. Consistently promoted both stocks, whereas NAG activity reduced SOC stock directly despite MBC-mediated mitigation. For TN stock, For TN, NAG’s negative direct effect was counterbalanced by strong positive indirect effects via MBC. Microbial biomass (MBC/MBN) emerged as the dominant factor (Figures 2d, 3d).

In MbF, the model explained 95% of the variance in both stocks (Figures 2e, 3e). NH₄^+^-N exerted direct and indirect positive effects. BG activity showed consistent positive effects on SOC stock but complex effects on TN stock. NAG activity again exhibited contrasting roles: indirect positive effects on SOC stock but direct negative effects on TN stock. Microbial biomass maintained strong direct relationships with both stocks (Figures 2f, 3f).

### 3.6 Drivers of soil organic carbon and total nitrogen stocks across soil depths

SOC and TN stocks showed distinct vertical stratification patterns. The SEM revealed key drivers of SOC and TN stocks across soil depths, with all models showing good fit (GFI ≥ 0.91, SRMR ≤ 0.040, *p* > 0.05).

**Figure 4:**
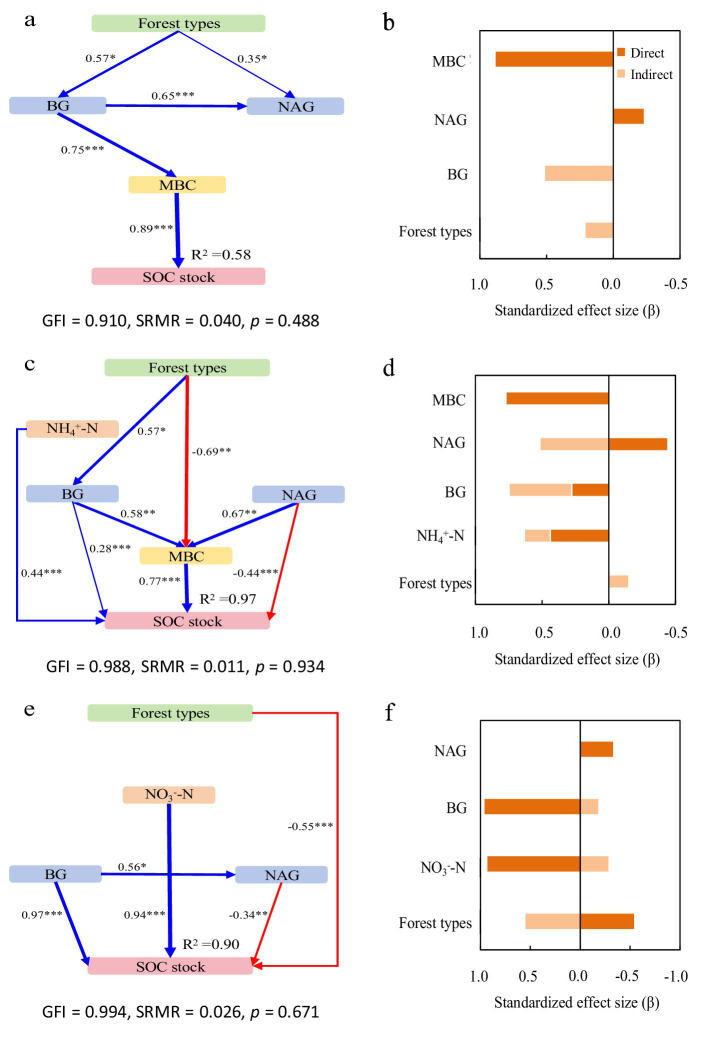
Structural equation models depicting the drivers of soil organic carbon (SOC) stocks in the 0–20 cm **(a,b)**, 20–40 cm **(c,d)**, and 40–60 cm **(e,f)** layers. Explanatory variables include ammonium nitrogen (NH_4_^+^-N), nitrate nitrogen (NO_3_^−^-N), β-glucosidase (BG) and N-acetyl-glucosaminidase (NAG), microbial biomass carbon (MBC) and microbial biomass nitrogen (MBN). **(a,c,e)** Pathways analysis of SOC stocks. The figures adjacent to the arrows represent path coefficients and their corresponding *p* values. The symbols *, **, and *** denote significance levels of *p* < 0.05, *p* < 0.01, and *p* < 0.001, respectively. **(b,d,f)** Summed the direct and indirect effects (Bars are not stacked, adjacent bars represent distinct effect types).

**Figure 5:**
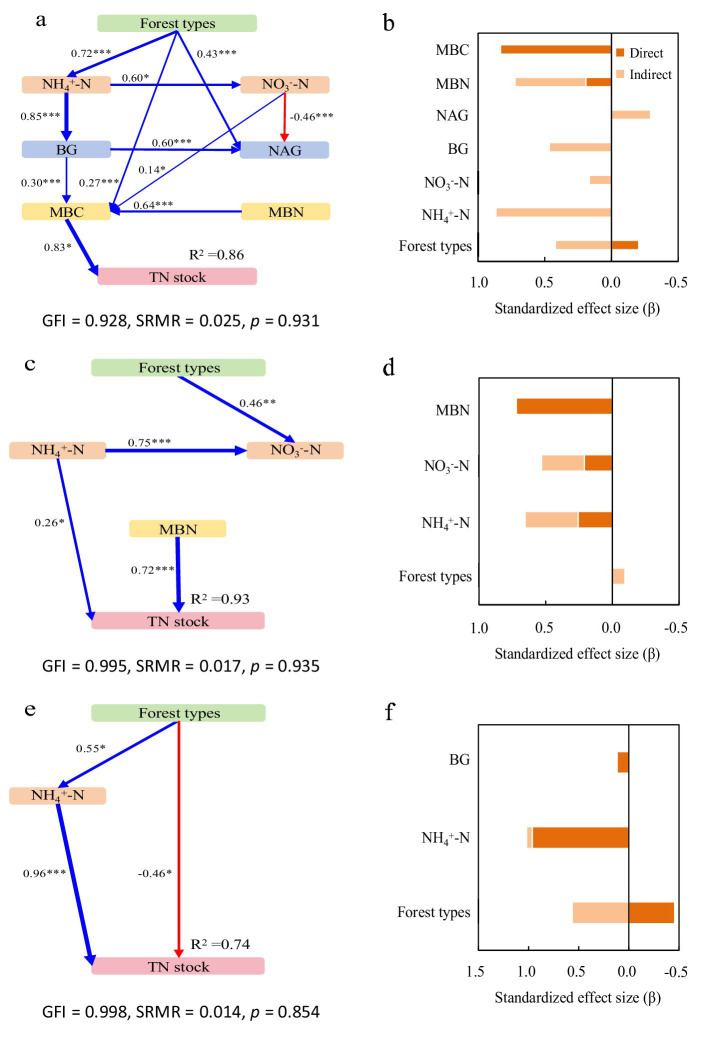
Structural equation models depicting the drivers of total nitrogen (TN) stocks in the 0–20 cm **(a,b)**, 20–40 cm **(c,d)**, and 40–60 cm **(e,f)** layers. Explanatory variables include ammonium nitrogen (NH_4_^+^-N), nitrate nitrogen (NO_3_^−^-N), β-glucosidase (BG) and N-acetyl-glucosaminidase (NAG), microbial biomass carbon (MBC) and microbial biomass nitrogen (MBN). **(a,c,e)** Pathways analysis of TN stocks. The figures adjacent to the arrows represent path coefficients and their corresponding *p* values. The symbols *, **, and *** denote significance levels of *p* < 0.05, *p* < 0.01, and *p* < 0.001, respectively. **(b,d,f)** Summed the direct and indirect effects (Bars are not stacked, adjacent bars represent distinct effect types).

In the surface layer (0–20 cm), the model explained 58 and 86% of variance in SOC and TN stocks, respectively (Figures 4a, 5a). Forest types increased both stocks indirectly through inorganic N, enzyme activities, and microbial biomass, while directly reducing TN stock. BG activity enhanced SOC stock via microbial pathways, whereas NAG activity directly decreased SOC and indirectly reduced TN stock. MBC was the strongest direct positive driver for both stocks (Figures 4b, 5b).

In the subsurface layer (20–40 cm), the model accounted for 97 and 93% of variance in SOC and TN stocks, respectively (Figures 4c, 5c). NH₄^+^-N showed direct and indirect positive effects on both stocks. NO₃^−^-N also contributed positively to TN. BG activity increased SOC stock through direct and indirect pathways, while NAG activity directly suppressed it—though partially offset by an indirect positive effect via MBC. Microbial biomass remained the dominant factor influencing both SOC and TN stocks (Figures 4d, 5d).

In the deeper layer (40–60 cm), the model explained 90 and 74% of variance in SOC and TN stocks, respectively (Figures 4e, 5e). Forest types directly reduced both stocks, but also exerted positive indirect effects. NH₄^+^-N was the primary direct driver of TN stock, while NO₃^−^-N strongly promoted SOC stock. BG activity continued to enhance SOC stock directly, and NAG significantly reduced it. Microbial biomass showed no significant influence at this depth (Figures 4f, 5f).

## 4 Discussion

### 4.1 Forest types effects on soil organic carbon and total nitrogen stocks

Contrary to our initial hypothesis, MbB and MbF did not show significantly higher SOC stocks than Mb stands (0–100 cm profile; Figure 1a). This suggests that tree species diversity alone may not enhance SOC accumulation, likely due to counteracting mechanisms between decomposition and stabilization processes, suggesting limited benefits of tree diversity for SOC accumulation. This aligns with subtropical region in southern China studies where opposing decomposition and stabilization processes neutralized diversity effects (Du et al., 2010; Qi et al., 2013). In MbB, broadleaved litter significantly enhanced N availability and stimulated enzyme activities, supporting the notion that litter quality strongly regulates microbial function (Stone et al., 2012). Despite these stimulatory effects, microbial biomass remained similar across forest types, suggesting faster turnover rates rather than increased standing biomass (Kuzyakov et al., 2000; Lajtha et al., 2014). The dual role of NAG—directly reducing SOC yet indirectly supporting it through microbial pathways—exemplifies the complex regulatory mechanisms that maintain SOC stability across systems. NAG activity directly reduced SOC but indirectly increased it via MBC (Figure 2). This probably because higher NAG activity may relieve microbial N limitation (Mooshammer et al., 2014). As a result, microbes can use more C, which temporarily reduces SOC. At the same time, more MBC contributes microbial residues to the soil, which can eventually increase SOC (Fan et al., 2021). This balance between positive (NH₄^+^-N, NO₃^−^-N, enzymes, MBC) and negative (enzymes) drivers maintained similar SOC stocks across forest types, masking potential differences in C cycling processes.

In contrast to SOC, TN stocks varied significantly among forest types, with MbF showing 32–34% lower stocks than Mb and MbB (Figure 1b). This pattern aligns with differences in microbial N retention across forest types, as evidenced by the significantly weaker microbial biomass N and enzyme activities in MbF (Figure 3). The direct negative relationship between of NAG activity and TN stocks (Figure 3f) enhanced microbial N mining from soil organic matter under conditions of N limitation, a response consistent with previously documented microbial strategies (Craine et al., 2007; Prescott, 2010; Liu et al., 2021). In contrast, MbB exhibited higher TN stocks, which may be partly attributed to its greater NH₄^+^-N availability (47% higher than Mb; Figure 1c) and stronger microbial N retention capacity (Figure 3c). The reduced synchronization between microbial biomass and enzyme activities in MbF likely lowered its nitrogen stabilization efficiency compared to the more tightly coupled system in MbB (Liang et al., 2017; Zhang et al., 2020). While NAG showed direct negative effects on TN in MbB, its indirect positive effects through microbial biomass compensation created an overall weakly positive influence. These results emphasize that variations in N cycle processes—particularly microbial retention and enzyme activities—can lead to substantial differences in TN accumulation across forest types. While litter properties are known to influence N dynamics, the mechanistic inferences drawn in this discussion are based primarily on the microbial and enzymatic parameters measured in our study. Further investigation incorporating direct measures of litter chemistry and microbial community composition would help clarify the drivers of the observed patterns.

SOC and TN stocks showed consistent positive relationships with microbial biomass (MBC, MBN) across all forest types (Figures 2, 3). The MbB system demonstrated optimized Changes in SOC and TN stocks through enhanced enzyme activities and microbial efficiency. These patterns support established findings that forest composition regulates enzyme-mediated decomposition, with broadleaved systems typically showing greater investment in hydrolytic enzymes (Sinsabaugh et al., 2008; Li et al., 2018). Notably, NO₃^−^-N showed divergent roles among forest types, with significant indirect effects only in MbB, reflecting niche differentiation in N cycling pathways (Elser et al., 2000; Högberg et al., 2006; Kooch et al., 2017; Wang et al., 2022). The significantly lower TN stocks in MbF (Figure 1b) highlight Chinese fir’s strong N uptake capacity through its extensive root system (Peng et al., 2021). These findings emphasize that while SOC stocks remained similar across forest types, tree species selection critically influences N dynamics in mixed forest management.

### 4.2 Depth-specific controls on soil organic carbon and total nitrogen stocks

Our findings reveal distinct depth stratification in forest-type effects, with strongest influences in surface soils (0–20 cm) where litter inputs and root activity dominate biogeochemical processes. While MbF and MbB showed the highest SOC and TN stocks in surface layers - consistent with the surface accumulation hypothesis (Jobbágy and Jackson, 2000) - no significant differences emerged among forest types for these stocks despite substantial variations in process rates. The structural equation models revealed a complex balance of direct and indirect effects: A direct negative association between forest type and TN stock was offset by positive indirect effects, resulting in a net weak positive association (Figure 5b). Notably, MbB exhibited substantially greater NH₄^+^-N (+125%) and NO₃^−^-N (+44%) concentrations (Figures 1c,d), BG activity (+75%; Figure 1e), and MBC (+50%; Figure 1g) compared to Mb, yet maintained comparable SOC/TN stocks. This paradox may reflect a system in which high N availability supports greater microbial biomass and enzyme production, leading to more efficient processing of organic matter. In such systems, enhanced microbial activity can simultaneously stimulate mineralization and promote stabilization through the formation of microbial necromass and organo-mineral associations (Liang et al., 2017; Prescott and Grayston, 2013; Six et al., 2006). Key enzymatic controls emerged: BG indirectly increased stocks via microbial pathways; NAG directly reduced SOC while indirectly decreasing TN; MBC showed strong positive associations with both SOC and TN stocks (Figures 4, 5). The indirect increase in SOC via BG activity implies that communities invested in substrate acquisition may simultaneously promote the production of microbial necromass and metabolites that are precursors for stable SOC formation. Conversely, the direct negative effect of NAG on SOC suggests that N-limiting conditions may trigger microbial mining of organic matter for N, inadvertently leading to SOC mineralization.

The 20–40 cm layer exhibited distinct biogeochemical patterns, with Mb showing significantly higher SOC and TN stocks than MbB and MbF (FIgures 1a,b). This vertical redistribution likely results from two key mechanisms: production of recalcitrant bamboo litter that decomposes slowly, enabling downward leaching of dissolved organic matter; deep root systems facilitating C and N translocation (Song et al., 2017; Liu M. et al., 2019, Liu X. et al., 2019). MbF displayed particularly low stocks in this layer: 42–48% lower SOC and TN than Mb; 34–48% lower than MbB. MbF displayed particularly low NH₄^+^-N concentrations in this layer: 52% lower than Mb and 44% lower than MbB (Figure 1c), alongside significant positive NH₄^+^-N effects on stocks (Figures 4d, 5d). Structural equation modeling revealed forest type indirectly suppressed stocks via inorganic N availability, enzyme activities, and microbial pathways (Figures 4d, 5d). The patterns suggest Chinese fir’s growth strategy prioritizes aboveground allocation over rhizosphere investment, as evidenced by lower root N content compared to bamboo, positive root N-soil N correlations (Peng et al., 2021), and reduced microbial-driven accumulation (Sinsabaugh et al., 2008). These findings highlight how plant functional strategies mediate vertical C-N distribution through coupled above-belowground allocation patterns.

No significant differences in SOC and TN stocks were detected among the 40–60 cm, 60–80 cm, and 80–100 cm layers across forest types, except for a 41% lower TN stock in MbF compared to MbB (Figure 1b). Structural equation modeling for the 40–60 cm layer revealed complex interactions: while forest type showed direct negative effects on SOC and TN stocks, these were nearly offset by positive indirect effects, resulting in weak net associations (Figures 4e,f, 5e,f). Inorganic N forms and BG activity emerged as key drivers of subsoil C and N stocks. NH₄^+^-N strongly influenced TN, consistent with its dominance in anaerobic subsoil environments where nitrification is limited (Huygens et al., 2007). Conversely, NO₃^−^-N showed a robust positive effect on SOC, likely through promoting iron-oxide mineral formation that stabilizes organic C in these acidic soils (Kleber et al., 2021). These patterns highlight the shift from biological to physicochemical controls on C and N stabilization in subsoil layers where enzyme activity is energy-limited (Rumpel and Kögel-Knabner, 2011).

Our results revealed distinct depth-dependent patterns in SOC and TN stock determinants. Consistent with the fact that bamboo fine roots are predominantly concentrated within 0–60 cm, microbial biomass dominated as the primary controlling factor in surface layers (0–40 cm), with MBC showing strong positive effects on SOC (0–20 cm) and TN stocks (0–20 cm). MBN significantly influenced TN stocks in the 20–40 cm layer (Figures 4, 5). BG activity exhibited contrasting depth patterns, directly enhancing SOC stocks in subsurface layers (20–60 cm) while only indirectly affecting surface SOC (0–20 cm). Notably, reduced SOC and TN stocks in MbF correlated strongly with NH₄^+^-N concentrations, particularly in the 20–40 cm layer where NH₄^+^-N showed direct and indirect effects on SOC (Figures 4c,d). The influence of NH₄^+^-N on TN stocks intensified with depth (20–60 cm), while microbial biomass effects diminished (Figures 4, 5). These observed patterns correspond to the unique root architecture of *Phyllostachys edulis*—a shallow-rooted species characterized by a high concentration (>80%) of fine roots within the upper 0–60 cm soil depth (Yang et al., 2021; Ni and Su, 2024). Consequently, significant biogeochemical differentiation occurred primarily above 60 cm, consistent with established depth gradients in soil C stability (Jobbágy and Jackson, 2000). Below this threshold, the convergence of SOC/TN controlling factors across forest types reflects the diminishing biological influence beyond the rhizosphere zone.

## 5 Conclusion

This study demonstrates that forest type significantly influences soil C and N dynamics, with a stronger effect on N stocks. Mixed forests did not increase SOC stocks compared to pure Moso bamboo forests (Mb). However, the mixed Moso bamboo-Chinese fir forests (MbF) exhibited significantly lower total nitrogen (TN) stocks (32–34%) than both Mb and mixed Moso bamboo–evergreen broadleaved forests (MbB), indicating a pronounced N limitation in coniferous-dominated systems. Depth-stratified analyses revealed contrasting controls: microbial biomass was the key regulator of C and N in surface soils (0–60 cm), while subsoils (> 60 cm) were dominated by physicochemical stabilization. The MbB stand maintained a C-N equilibrium, whereas MbF was characterized by enzymatic N mining and reduced microbial nutrient retention. We conclude that tree species composition is a critical driver of soil N retention, and broadleaved species should be favored in mixed plantations to enhance ecosystem N cycling and storage. Future research should target the long-term legacy of tree species on subsurface soil processes.

## Data Availability

The original contributions presented in the study are included in the article/Supplementary material, further inquiries can be directed to the corresponding author.
