# Supplementary material for: Depth-dependent stabilization mechanisms of soil organic carbon and total nitrogen in different mixed modes of subtropical Moso bamboo forests
Source: Front Microbiol. 2025 Nov 12;16:1671811. doi: 10.3389/fmicb.2025.1671811 (PMC12647007; doi:10.3389/fmicb.2025.1671811)
Supplement: Supplementary file 1 [file Data_Sheet_1.docx]

**Depth-dependent stabilization mechanisms of soil organic carbon and total nitrogen in different mixed modes of subtropical Moso bamboo forests**

**Table S1.** Stand characteristics of the three forest types. The mean values ±1 SE of diameter at breast height (DBH) and stand density are shown.

| Forest types code | Forest types | Species | Diameter (cm) | Stand density (trees/hm^2^) | Age(yr) |
| --- | --- | --- | --- | --- | --- |
| Mb | pure Moso bamboo forest | Moso bamboo | 9.92(±0.30) | 2700(±535) | 3 - 5 |
| MbB | mixed Moso bamboo–evergreen broadleaved forest | Moso bamboo | 8.96(±0.18) | 3300(±361) | 3 - 5 |
|  |  | *Castanopsis sclerophylla* | 7.83(±1.88) | 300(±58) | 10-15 |
|  |  | *Quercus glauca* | 10.20(±0.73) | 467(±67) | 10-15 |
| MbF | mixed Moso bamboo-Chinese fir forest | Moso bamboo | 11.10(±0.25) | 1433(±437) | 3 - 5 |
|  |  | Chinese fir | 10.66(±0.41) | 2067(±371) | 10-15 |

**Table S2.** Relationships between SOC, TN and forest types, soil depth, soil moisture content, NH_4_^+^-N, NO_3_^−^-N, β-glucosidase, N-acetyl-glucosaminidase, microbial biomass carbon and microbial biomass nitrogen (n=60).

|  | SOC | TN | FT | SD | NH4^+^-N | NO3^-^-N | BG | NAG | MBC | MBN |
| --- | --- | --- | --- | --- | --- | --- | --- | --- | --- | --- |
| SOC | 1.000 |  |  |  |  |  |  |  |  |  |
| TN | 0.964** | 1.000 |  |  |  |  |  |  |  |  |
| FT | -0.008 | 0.017 | 1.000 |  |  |  |  |  |  |  |
| SD | -0.761** | -0.690** | 0.000 | 1.000 |  |  |  |  |  |  |
| NH4^+^-N | 0.712** | 0.743** | 0.231 | -0.632** | 1.000 |  |  |  |  |  |
| NO3^-^-N | 0.575** | 0.560** | 0.221 | -0.446** | 0.718** | 1.000 |  |  |  |  |
| BG | 0.766** | 0.768** | 0.259* | -0.710** | 0.746** | 0.503** | 1.000 |  |  |  |
| NAG | 0.668** | 0.635** | 0.286* | -0.747** | 0.753** | 0.436** | 0.871** | 1.000 |  |  |
| MBC | 0.839** | 0.815** | 0.107 | -0.742** | 0.785** | 0.649** | 0.804** | 0.808** | 1.000 |  |
| MBN | 0.838** | 0.834** | -0.069 | -0.713** | 0.698** | 0.547** | 0.711** | 0.681** | 0.945** | 1.000 |

** highly significant (P < 0.01). * significant (P < 0.05).

FT: forest types.

SD: soil depth.

BG: β-glucosidase.

NAG: N-acetyl-glucosaminidase.

MBC: microbial biomass carbon.

MBN: microbial biomass nitrogen.
